# Utilization of tea grounds as feedstuff for ruminant

**DOI:** 10.1186/2049-1891-4-54

**Published:** 2013-12-26

**Authors:** Huili Wang, Chuncheng Xu

**Affiliations:** 1 College of Engineering, China Agricultural University, Beijing 100083, P. R. China

**Keywords:** Ensiling, Fermentation quality, Nutritive value, Tea grounds, Total mixed ration

## Abstract

Researches on tea have been developed for decades, which prove that tea, especially green tea, has multiple functional components. With the rapid development of beverage industry, the resultant large amounts of tea grounds attract great attention. However, unreasonable utilization of tea grounds would lead to great waste and environmental pollution, especially in summer. In view of the high nutritive value and multiple functional components, tea grounds could be used as feedstuff. By now, researches of tea grounds as feedstuff are mainly on ruminant, as the utilization on other animals is limited to lower level due to high fiber content. Therefore, the following review will begin with a brief introduction of tea grounds and the possible utilization of tea grounds as feedstuff, and then elaborate on the application of ensiling and total mixed ration on ruminant. Apart from the fermentation quality, nutritive value is also provided to assess feasibilities of using tea grounds as feedstuff resources. Finally, a summary on the utilization situation and future direction of using tea grounds as feedstuff is provided in this review.

## Introduction

Tea is one of the most popular beverages in the world [1,2]. With the increasing awareness on health and nutrition issues in beverage market, consumption of tea drinks, especially green tea has been increasing significantly in recent years. Accompanied with the consumption of ready-made tea drinks in bottles, packs and cans, a large amount of tea grounds are released annually by beverage companies. Although only small amounts of tea grounds are converted into raw compost material, most are generally buried [3]. In view of the high nutritive value and multiple functional components, tea grounds could be used as feed resources or nutrient supplementary. However, unreasonable utilization of tea grounds could lead to great waste and environmental pollution, especially in summer. There is increasing demand for efficient use of food by-products due to economic and environmental concerns [4].

Nowadays in China, utilization of wet or dry tea grounds on the production of chickens and pigs has been reported, which indicates that the addition of tea grounds at proper amount could enhance or maintain the quality and output of the resultant meat and eggs [5,6]. However, high fiber content restricts the proportion of tea grounds in diet. While for ruminant, there is no such problem but how to make efficient utilization and obtain better animal performance. Therefore, researches published recently focus on the effective utilization of tea grounds on ruminant.

### Chemical composition and feed characteristics

Tea grounds are mainly by-products of ready-made tea drinks, which are derived from tea leaves extracted by hot water to make tea drinks. Tea leaf has a useful content of amino acids, proteins, vitamins, tannins and polyphenols [7]. The catechins are present in higher quantities in green tea than in black or oolong tea, because of different processing of tea leaves after harvest [1]. It has been indicated that polyphenols especially the flavonoids exhibit anticarcinogenic, antimutagenic, and cardioprotective effects which are generally associated with their antioxidant (free radical scavenging and metal chelation) properties [2,8].

However, most of these nutrients remain in tea grounds. After extraction by hot water, green tea grounds (GTG) still contain a lot of protein, tannin, caffeine, beta-carotene and vitamin E [9], which might help to prevent disease if fed to ruminants. Total extractable tannin and condensed tannin (CT) contents are higher in GTG than in black tea and oolong tea grounds [10]. According to the previous studies [3,10-12], GTG usually contain 22-35% of crude protein (CP), 2.3-7.1% ether extract (EE), 24-37% acid detergent fiber (ADF) and 31-45% neutral detergent fiber (NDF) on dry matter (DM) basis. Whereas, barley tea grounds (BTG) contain 12-19% of CP, 2.2-3.4% EE, 15-25% ADF and 27-35% NDF on DM basis [13-15]. All these suggest that tea grounds have a potential as a feed resource.

However, the high moisture content makes it deteriorated easily after being released by beverage companies, especially in summer. Therefore, it must be used as soon as possible if fed directly. However, direct-fed exhibits poor palatability due to the tannin components existed in tea grounds. Furthermore, it can be used as dry feed due to its high nutrient, except that drying process would consume a lot of energy. Considering the above characteristics, ensiling could be one of the suitable ways to preserve high-moisture tea grounds.

### Silage preparation and fermentation quality

Nutrient losses and proteolysis are inevitable during the ensiling process. They are always reduced or inhibited by a rapid decline in pH during the initial period to create a low pH environment that is unsuitable for the action of plant and microbial proteases [16,17].

Preservation by ensiling is highly dependent on lactic acid fermentation. Whereas, tea grounds usually contain about 10^6^ aerobic bacteria, 10^3^ to 10^4^ mold and yeast in colony forming units/g of fresh matter, whereas lactic acid bacteria (LAB) could not be detected. Besides, water-soluble carbohydrates (WSC) are consistently at or below the detectable level [9,12]. Thereby, lactic acid fermentation scarcely occurs when untreated tea grounds are ensiled alone. In order to solve such problems, many researches have been carried out by adding additives or mixed ensiled with other feed resources.

#### ***Fermentation quality of tea grounds silage***

Tea grounds are usually ensiled with additives or in mixed silage with other materials. Researches indicated that addition of *Lactobacillus plantarum* and commercial acremonium cellulase (AUS) in both GTG and BTG could improve the fermentation quality as indicated by lower pH and NH_3_-N content and higher lactic acid content [9,12,18]. It is probably that the inoculation ensures sufficient LAB at the initial period, while AUS increased WSC content, which could be used by LAB to produce lactic acid. BTG silage could also be well preserved when treated by formic acid or sodium hydroxide (NaOH) which could inhibit even the fermentation of LAB, and therefore, exhibited low NH_3_-N content, no lactic acid, acetic acid, propionic acid and butyric acid [18]. Wang et al. [3] evaluated the fermentation quality of GTG silage treated with LAB, enzyme, formic acid and formaldehyde, and suggested that LAB treatment had the best effect on improving fermentation quality and inhibiting protein degradation.

In addition, it has been found that the inclusion of high-quality materials in GTG silage may be an effective way to improve fermentation quality. GTG could be ensiled successfully without bacterial inoculants when mixed with materials containing sufficient sugars [19,20].

Besides, it is also indicated that the addition of tea grounds could enhance the lactic acid fermentation of sudangrass and oat silage, which contains adequate WSC content. However, the fermentation quality differs by variety of tea and addition ratio [10,21,22]. Apart from forage silage, GTG could also enhance the lactic acid fermentation of byproducts-mixed silage when there are insufficient materials for lactic acid production [23]. However, research reveals that neither GTG-associated LAB nor green tea polyphenols could account for the enhancement of lactic acid fermentation. It is probably that GTG supply some nutrients other than polyphenols, which are heat-stable and effective for LAB growth during ensiling [22]. Further research is still required to clarify the mechanisms of enhanced lactic acid fermentation by the addition of GTG.

#### ***Fermentation quality of total mixed ration (TMR) silage***

Ensiling is suitable for preserving high-moisture tea grounds. However, there are problems with the use of tea grounds as feed, such as nutritional imbalance, poor palatability and poor preservation [24,25]. If ensiled with dry feeds as a TMR, the risk of effluent production would be minimized and the time for mixing prior to feeding could be omitted. In addition, unpalatable by-products could be incorporated into TMR if their odors and flavors could be altered by silage fermentation [4].

Noor et al. [26] reported that GTG could be used as an ingredient in TMR silage production for animal feeding. However, it is suggested that the fermentation period should not exceed 3 mo if ensiled in a flexible container bag silo, when some decrease in quality would occurred. Xu et al. [24,25] reported that the addition of GTG and BTG at 10%, 20% and 30% DM ratio of TMR could all be well preserved and exhibited low pH and NH_3_-N content, and high lactic acid content. Suto et al. [27] recommended that the moisture content of TMR should be held at about 55%, when the TMR contains 10-30% of GTG on DM basis, as the lactic acid content tended to increase with the proportion of GTG increased and with the moisture content of the TMR decreased.

Besides, many researches have shown that the substitution of GTG for wet brewers’ grains (BG) at ratios of up to 15% on DM basis of TMR could be well preserved with high lactic acid content, low pH and NH_3_-N content [4,28]. While, the replacement of BTG and soybean meal mixture (7:3 on DM basis) for BG at ratios of up to 15% DM of TMR could increase lactic acid concentration, decrease pH, acetic acid and NH_3_-N of the TMR silage [15].

### Nutritive value of tea grounds silage and TMR silage

In addition to fermentation characteristics of silage, their nutritive value for ruminant also need to be evaluated, which is assessed by feed intake, digestibility, rumen fermentation, nitrogen (N) balance, blood component and milk production.

#### ***Nutritive value of tea grounds silage***

Many researches have assessed the nutritive value of fresh tea grounds and tea grounds silage from different aspects.

The nutritive value of GTG is thought to be equivalent to that of BG. For GTG silage, digestibilities of CP and EE are 74.6% and 50.7% on DM basis, respectively; Besides, the estimated total digestible nutrients (TDN), digestible CP and digestible energy are 71.1%, 23.9% and 13.4 MJ/kg on DM basis, respectively [12]. For BTG silage, however, digestibilities of CP and organic cell wall are 26.8% and 30.2%, and the TDN, digestible CP, digestible energy are 65.1%, 4.8% and 13.0 MJ/kg on DM basis, respectively. It is suggested that the nutritive value of BTG silage is about 80% of that of barley [14,29]. Therefore, both GTG and BTG can be used as feed ingredient.

Xu et al. [18] reported that BTG silage treated with NaOH exhibited the highest degradability of DM and CP than that of the control and BTG, then it followed by the LAB + AUS treatment. However, formic acid treatment had no effect on the DM and CP disappearance of silage.

Many researches have indicated that well-fermented GTG silage contributes to preserve more tea catechins and antioxidative activity [12,20]. Nishino et al. [20] investigated the changes due to ensiling in tea catechins and antioxidative activity of wet GTG. Results indicated that ensiling significantly lowered antioxidative activity and decreased the contents of partial tea catechins in GTG silage, whereas, reductions of tea catechins were ameliorated and no marked changes were found in total phenols and antioxidative activity during ensiling when ensiled as a mixture with dried beet pulp. Inhibited degradation of tea catechins were also found in wet GTG silage treated with LAB and cell wall degrading enzymes [12].

Besides, it has been indicated that CT could reduce rumen forage protein degradation due to reversible binding to these proteins [30], suppress the breakdown of protein by rumen microorganisms [31] and decrease ruminal gas production [32]. It is consistent with the study of Nishino et al. [20] that addition of dried beet pulp increased gas production of GTG silage, which in other words suppressed gas production with the increase of GTG. However, the reduction of tea catechins during ensiling would not elicit an improvement in digestibility of GTG silage. It is consistent with the finding of Salawu et al. [31] that tannin protected proteolysis of proteins during silage fermentation but was digestible in the lower gut, and it also agreed with finding of Kondo et al. [21] that proteins in GTG seem to be stable during ensiling, but digestible post-ruminally.

Evidence also indicates that CT at levels of 20–45 g/kg DM can reduce rumen forage protein degradation and protect amino acids to increase the absorption in the small intestine of ruminants, while deteriorating intake and digestibility at >55 g/kg DM [20,30]. It may illustrate the following no detrimental effect on gas production with the increase of GTG, which probably due to the less amount of tannins than that is critical to suppress the activity of rumen bacteria. Kondo et al. [10] found that the addition of GTG to forage silage could increase gas production, whereas addition of oolong and black tea grounds suppressed gas production. It is agreed with Kondo et al. [23] that the addition of GTG to byproducts-mixture increased gas production.

Furthermore, many researches focused on the nutritive value of silage with GTG added in different ratio. Kondo et al. [21] studied the feeding value of oat silage with GTG added at a ratio of 0, 5% and 20% on fresh matter. No significant differences were found in DM intake and digestibility, except for the increased CP digestibility with the increment of GTG. In addition, N retention and ruminal NH_3_-N were increased, while total volatile fatty acids (VFA) were reduced by addition of GTG. The high CP content, N digestibility and N retention indicated the potential of GTG as a protein supplement, and the addition of GTG could be up to 20% on fresh matter in oat silage.

#### ***Nutritive value of tea grounds TMR silage***

Previous studies have shown that GTG can be used as protein supplements due to the high nutrition and low cost [9,12]. On the other hand, tannins exhibit contrasting effects on feed intake and digestibility when GTG applied in different ratio [30]. All these suggest that uncertain results may appear on the utilization of tea grounds. Thus, further researches are required to have a better understanding of the utilization of tea grounds.

Suto et al. [27] reported that the moisture content of TMR varied from 55% to 75% had small effects on the degradability and the effective degradability of TMR containing GTG at 10-30% on DM basis.

Besides, many researches have reported the feeding value of TMR silage with BTG in different application ratio. Xu et al. [25] reported that, digestibilities of DM, CP and TDN were significantly higher for TMR silages with 10% and 20% of BTG than that with 30% of BTG. Therefore, the ideal mixing proportion of BTG for TMR silage is 10% to 20% on DM basis. However, it was also reported that the possible proportion of replacing wet BG with BTG for TMR silage was suggested to be 10% or less of diet DM [15]. In the study, feed intake and digestibility for the 5% and 10% treatments were not different from the control. However, feed intake and digestibilities of EE and NDF were lower for the 15% treatment. In addition, the ruminal total VFA concentration was higher, while NH_3_-N content was lower for the 15% treatment compared with the control.

Furthermore, progressive increase of GTG (0, 5%, 10% and 15% on DM of TMR) substituted for wet BG had no effect on voluntary feed intake of TMR silage, while digestibility was slightly lower than the control. No differences among treatments were observed in retention N, pH and total VFA concentration [4]. The study suggested that a high GTG level of 15% of diet DM can be recommended for silage based TMR. In contrast, feed intake and N retention of TMR silage were also not affected with the GTG increased up to 15% [28]. Compared with the control, digestibilities for the 5% and 10% treatments were not different, while that for 15% treatment was lower. With the increase in GTG, N intake did not differ, but fecal N increased, while urinary N and plasma urea N content were decreased. However, ruminal NH_3_-N in the 15% treatment was lower than that of the control. Therefore, the possible mixing proportion of GTG for TMR silages can be 10% of the diet DM.

Kondo et al. [11] found that the DM intake of cows fed TMR with GTG silage was slightly but not significantly increased with the increment of GTG, while ensiled GTG contained high amounts of lactic acid, acetic acid and CT. It is inconsistent with the negative correlation between DM intake of silage and the acetic acid and lactic acid concentrations [33], or the decreased feed intake in ruminants owning to high tannin [34]. It is supposed that the inclusion of GTG silage at 5% DM in TMR were relatively low to show the negative impact of tannin on feed intake. Furthermore, GTG silage incorporated into TMR at a ratio of 5% on DM and 10% on CP basis had no detrimental effects on the performance of lactating cows. Therefore, the addition of GTG silage is suggested to be no less than 5% on DM basis of TMR, which is also consistent with the results of Eruden et al. [35] that addition of GTG silage at 5% of diet DM did not affect the performance of midlactating cows. However, Eruden et al. [36] reported that, considering that the feed intake was not affected, the upper limit of mixing ratio for GTG silage is expected to be 8 and 15% in TMR with timothy hay and maize silage as main roughage, respectively.

Eruden et al. [37,38] also studied the addition of GTG silage at 10% or 20% of diet DM. Compared with the control, no significant effects were found on feed intake and digestibility in both 10% and 20% tea group, except that the CP intake was increased and digestibilities of ADF and NDF were decreased in the 20% group. Besides, addition of GTG at 10% diet DM had no significant effect on ruminal fermentation, plasma metabolites, as well as milk yield and components. All the above indicated that GTG silage could be added up to 20% of diet DM. Furthermore, Nishida et al. [39] suggested that feeding diets containing 20% of GTG silage had no negative impact on ruminal fermentation, but increased the plasma antioxidative activity and vitamin E concentration.

## Conclusions

Ensiling is suitable for preserving tea grounds. Both ensiled with additives and mixed ensiled could be well preserved. Besides, adjusting moisture with dry feeds as a TMR is also effective to incorporate GTG as feedstuff. According to the previous researches, the addition of tea grounds in mixed silage could be up to 20% on fresh matter and the possible mixing proportion of tea grounds for TMR silage is suggested to be less than 10% or 10% to 20% on DM basis. Whereas, the inclusion of tea grounds silage for TMR is inconsistent and differs by the variety of main roughage, ranging from 5% to 15% or more on DM basis.

Apart from the possible addition ratios in silage and TMR, researches also provide feasibilities of using tea grounds as protein resources or substitution of other feed materials. The characteristics of high protein content and low cost will certainly relieve the dependence to some extent on imported protein products. However, there are also problems that GTG are always mass-produced and beverage companies are always scattered at suburbs. Therefore, it is necessary to work out a reasonable plan to reduce the unnecessary cost.

Furthermore, in view of the controversies on tea catechins and CT, it is necessary to have a further understanding of the functional ingredients in tea grounds and their association with animal performance, so as to make efficient use and obtain better performance. Besides, efforts should be made to reduce the high fiber content so as to reduce the limitation on other animals, as well as to assess the potential risk of tea grounds, such as pesticide residues.

## Abbreviations

ADF: Acid detergent fiber; BG: Brewers’ grains; BTG: Barley tea grounds; CP: Crude protein; CT: Condensed tannin; DM: Dry matter; EE: Ether extract; GTG: Green tea grounds; LAB: Lactic acid bacteria; N: Nitrogen; NDF: Neutral detergent fiber; TDN: Total digestible nutrients; TMR: Total mixed ration; VFA: Volatile fatty acids; WSC: Water-soluble carbohydrates.

## Competing interests

The authors declare that they have no competing interests.

## Authors’ contributions

HLW collected and sorted the related information and drafted the manuscript. CCX conceived the study, participated in its design and arrangement of the review, and helped to draft the manuscript. Both authors have read and approved the manuscript.

## Authors’ information

Huili Wang is a doctor candidate of College of Engineering, China Agricultural University, with expertise in the utilization and evaluation of non-conventional feed resources. Chuncheng Xu, Ph.D., is a professor of Animal Nutrition and Science in China Agricultural University, with expertise in research and development of non-conventional feed ingredients and nutrition evaluation for ruminants.

## References

[B1] Graham HN Green tea composition, consumption, and polyphenol chemistry Prev Med 1992 4 334 350 10.1016/0091-7435(92)90041-F 1614995

[B2] Khokhar S Magnusdottir S Total phenol, catechin, and caffeine contents of teas commonly consumed in the United Kingdom J Agric Food Chem 2002 4 565 570 10.1021/jf010153l 11804530

[B3] Wang RR Wang HL Liu X Xu CC Effects of different additives on fermentation characteristics and protein degradation of green tea grounds silage Asian-Australas J Anim Sci 2011 4 616 622 10.5713/ajas.2011.10346

[B4] Xu C Cai Y Moriya N Ogawa M Nutritive value for ruminants of green tea grounds as a replacement of brewers’ grains in totally mixed ration silage Anim Feed Sci Technol 2007 4 228 238 10.1016/j.anifeedsci.2006.11.014

[B5] Ma T Application prospect of tea polyphenols in feed Anim Breed Feed 2008 4 92 94 (In Chinese)

[B6] Gao F Tian K Wang J A study on the feeding value of quick-melting tea residue: 1. the residue’s feeding effect on fattening pigs J Hunan Agr Univ 1998 4 465 467 (In Chinese)

[B7] Yamamoto T Juneja LR Chu DC Kim M Chemistry and application of green tea 1997 Boca Raton, Florida: CRC Press

[B8] Dufresne CJ Farnworth ER A review of latest research findings on the health promotion properties of tea J Nutr Biochem 2001 4 404 421 10.1016/S0955-2863(01)00155-3 11448616

[B9] Cai Y Masuda N Fujita Y Kawamoto H Ando S Development of a new method for preparation and conservation of tea grounds silage Anim Sci J 2001 4 536 541

[B10] Kondo M Kita K Yokota H Effects of tea leaf waste of green tea, oolong tea, and black tea addition on sudangrass silage quality and *in vitro* gas production J Sci Food Agric 2004 4 721 727 10.1002/jsfa.1718

[B11] Kondo M Nakano M Kaneko A Agata H Kita K Yokota HO Ensiled green tea waste as partial replacement for soybean meal and alfalfa hay in lactating cows Asian-Australas J Anim Sci 2004 4 960 966

[B12] Xu C Cai Y Fujita Y Kawamoto H Sato T Masuda N Chemical composition and nutritive value of tea grounds silage treated with lactic acid bacteria and *Acremonium* cellulase Anim Sci J 2003 4 355 361 10.1046/j.1344-3941.2003.00126.x

[B13] Cai Y Fujita Y Sato T Masuda N Nishida T Ogawa M Preparation and conservation of barley tea grounds silage and its fermentation quality Anim Sci J 2002 4 283 289

[B14] Enishi O Tsukahara N Kajikawa H Terada F Analysis of *in situ* ruminal disappearance and nutritive value of barley water residue Anim Sci J 2000 4 252 257

[B15] Xu C Cai Y Fukasawa M Matsuyama H Moriya N The effect of replacing brewers’ grains with barley tea grounds in total mixed ration silage on feed intake, digestibility and ruminal fermentation in wethers Anim Sci J 2008 4 575 581 10.1111/j.1740-0929.2008.00566.x

[B16] McDonald P Henderson AR Heron SJE The Biochemistry of Silage 1991 2 Marlow, UK: Chalcombe Publications

[B17] Rooke JA Hatfield RD Buxton DR, Muck RE, Harrison HJ Biochemistry of ensiling Silage Science and Technology 2003 Madison, Wisconsin: American Society of Agronomy 95 139

[B18] Xu C Cai Y Zhang H Fukasawa M Moriya N Ensiling and subsequent ruminal degradation characteristics of barley tea grounds treated with contrasting additives Anim Feed Sci Technol 2008 4 368 374 10.1016/j.anifeedsci.2007.05.032

[B19] Cai Y Fujita Y Xu C Ogawa M Sato T Masuda N Mixed silage preparation of green tea grounds and corn and its fermentation quality Anim Sci J 2003 4 203 211

[B20] Nishino N Kawai T Kondo M Changes during ensilage in fermentation products, tea catechins, antioxidative activity and *in vitro* gas production of green tea waste stored with or without dried beet pulp J Sci Food Agric 2007 4 1639 1644 10.1002/jsfa.2842

[B21] Kondo M Kita K Yokota H Feeding value to goats of whole-crop oat ensiled with green tea waste Anim Feed Sci Technol 2004 4 71 81 10.1016/j.anifeedsci.2003.10.018

[B22] Kondo M Naoki N Kazumi K Yokota H Enhanced lactic acid fermentation of silage by the addition of green tea waste J Sci Food Agric 2004 4 728 734 10.1002/jsfa.1726

[B23] Kondo M Kita K Yokota HO Evaluation of fermentation characteristics and nutritive value of green tea waste ensiled with byproducts mixture for ruminants Asian-Australas J Anim Sci 2006 4 533 540

[B24] Xu C Cai Y Kida T Matsuo M Kawamoto H Murai M Silage preparation of total mixed ration with green tea grounds and its fermentation quality and nutritive value Grassl Sci 2004 4 40 46

[B25] Xu C Cai Y Murai M Fermentation quality and nutritive value of total mixed ration silage with barley tea grounds Anim Sci J 2004 4 185 191

[B26] Noor RM Cai Y Uegaki R Matsuyama H Xu C Yoshida N Tando Y Miwa T Xie H, Huang J Fermentation quality of TMR silage with green tea grounds ensiled in flexible container bag silo XXI International Grassland Congress and VIII International Rangeland Congress; July 2008 2008 Hohhot, China: Guangdng People’s Publishing House 733

[B27] Suto R Horiguchi K Takahashi T Toyokawa K Effect of mixing proportion of green tea waste and moisture content on the fermentation quality and the rate of *in situ* degradation of TMR silage JPN J Grassl Sci 2007 4 127 132

[B28] Xu C Cai Y Moriya N Eruden B Hosoda K Matsuyama H Influence of replacing brewers’ grains with green tea grounds on feed intake, digestibility and ruminal fermentation characteristics of wethers Anim Sci J 2008 4 226 233 10.1111/j.1740-0929.2008.00521.x

[B29] Xu C Cai Y Fujita Y Kawamoto H Sato T Masuda N Silage preparation of barley tea grounds and their nutritive value Anim Sci J 2003 4 343 348

[B30] Min B Barry T Attwood G Mcnabb W The effect of condensed tannins on the nutrition and health of ruminants fed fresh temperate forages: a review Anim Feed Sci Technol 2003 4 3 19 10.1016/S0377-8401(03)00041-5

[B31] Salawu M Acamovic T Stewart C Hvelplund T Weisbjerg MR The use of tannins as silage additives: effects on silage composition and mobile bag disappearance of dry matter and protein Anim Feed Sci Technol 1999 4 243 259 10.1016/S0377-8401(99)00105-4

[B32] Makkar HP Blümmel M Becker K *In vitro* effects of and interactions between tannins and saponins and fate of tannins in the rumen J Sci Food Agric 1995 4 481 493 10.1002/jsfa.2740690413

[B33] Jones G Larsen R Lanning N Prediction of silage digestibility and intake by chemical analyses or in vitro fermentation techniques J Dairy Sci 1980 4 579 586 10.3168/jds.S0022-0302(80)82974-2 7381081

[B34] Silanikove N Nitsan Z Perevolotsky A Effect of a daily supplementation of polyethylene glycol on intake and digestion of tannin-containing leaves (*Ceratonia siliqua*) by sheep J Agric Food Chem 1994 4 2844 2847 10.1021/jf00048a035

[B35] Eruden B Nishida T Matsuyama H Hosoda K Shioya S Effect of the addition of various levels of green tea grounds silage at on the feed intake and milk production in lactating dairy cows Anim Sci J 2005 4 295 301

[B36] Eruden B Nishida T Matsuyama H Hosoda K Shioya S Nonaka K Yamada A Effect of main roughages on the palatability by lactating dairy cows of total mixed rations with green tea waste silage JPN J Grassl Sci 2006 4 232 236

[B37] Eruden B Nishida T Hosoda K Shioya S Cai Y Effects of green tea grounds silage on digestibility, rumen fermentation, and blood components in lactating dairy cows Anim Sci J 2003 4 483 490 10.1046/j.1344-3941.2003.00142.x

[B38] Eruden B Nishida T Hosoda K Shioya S Nutritive value of green tea grounds silage and influence of polyethylene glycol on nitrogen metabolism in steers Anim Sci J 2004 4 559 566

[B39] Nishida T Eruden B Hosoda K Matsuyama H Nakagawa K Miyazawa T Shioya S Effects of green tea (*Camellia sinensis*) waste silage and polyethylene glycol on ruminal fermentation and blood components in cattle Asian-Australas J Anim Sci 2006 4 1728 1736

